# Isolation of derivatives from the food-grade probiotic *Lactobacillus johnsonii* CNCM I-4884 with enhanced anti-*Giardia* activity

**DOI:** 10.1080/19490976.2025.2474149

**Published:** 2025-03-27

**Authors:** Anne-Sophie Boucard, Saulius Kulakauskas, Jana Alazzaz, Soraya Chaouch, Mohamed Mammeri, Aaron Millan-Oropeza, Carine Machado, Céline Henry, Christine Péchoux, Holger Richly, Michael Gassel, Philippe Langella, Bruno Polack, Isabelle Florent, Luis G. Bermúdez-Humarán

**Affiliations:** a Département Adaptation du Vivant, Université Paris-Saclay, INRAE, AgroParisTech, Micalis Institute, Jouy-en-Josas, France; b UMR 7245, Muséum National d’Histoire Naturelle, Centre National de la Recherche Scientifique, Sorbonne Universités, Paris, France; c Anses, INRAE, Ecole Nationale Vétérinaire d’Alfort, UMR BIPAR, Laboratoire de Santé Animale, Maisons-Alfort, France; d Plateforme d’Analyse Protéomique Paris Sud-Ouest (PAPPSO), INRAE, MICALIS Institute, Université Paris-Saclay, Jouy-en-Josas, France; e Université Paris-Saclay, INRAE, AgroParisTech, GABI, Jouy-en-Josas, France; f Boehringer Ingelheim Vetmedica GmbH, Kathrinenhof Research Center, Rohrdorf, Germany

**Keywords:** *Lactobacillus johnsonii*, probiotic, *Giardia intestinalis*, giardiasis, Bile salt hydrolases

## Abstract

Giardiasis, a widespread intestinal parasitosis affecting humans and animals, is a growing concern due to the emergence of drug-resistant strains of *G. intestinalis*. Probiotics offer a promising alternative for preventing and treating giardiasis. Recent studies have shown that the probiotic *Lactobacillus johnsonii* CNCM I-4884 inhibits *G. intestinalis* growth both *in vitro* and *in vivo*. This protective effect is largely mediated by bile salt hydrolase (BSH) enzymes, which convert conjugated bile acids (BAs) into free forms that are toxic to the parasite. The objective of this study was to use adaptive evolution to develop stress-resistant derivatives of *L. johnsonii* CNCM I-4884, with the aim of improving its anti-*Giardia* activity. Twelve derivatives with enhanced resistance to BAs and reduced autolysis were generated. Among them, derivative M11 exhibited the highest *in vitro* anti-*Giardia* effect with enhanced BSH activity. Genomic and proteomic analyses of M11 revealed two SNPs and the upregulation of the global stress response by SigB, which likely contributed to its increased BAs resistance and BSH overproduction. Finally, the anti-*Giardia* efficacy of M11 was validated in a murine model of giardiasis. In conclusion, our results demonstrate that adaptive evolution is an effective strategy to generate robust food-grade bacteria with improved health benefits.

## Introduction

*Giardia intestinalis* is the protozoan responsible for giardiasis, a widespread intestinal infection affecting humans and animals.^1^ Acute giardiasis is characterized by intestinal malabsorption, diarrhea, abdominal pain, nausea, vomiting and weight loss. In humans, giardiasis can lead to post-infectious complications, such as irritable bowel syndrome (IBS), chronic fatigue and growth retardation.^2–4^ Treatment of *G. intestinalis* infection usually consists of nitroimidazoles or benzimidazoles. However, failure rates range from 5% to 50%, and resistance to these drugs has increased in the last 15 years.^5–7^ Alternative strategies to control *G. intestinalis* infections include the repurposing of approved drugs and structural modifications of nitroimidazoles and benzimidazoles,^5,8^ the use of polyphenols derived from medicinal plants, including ginger and curcumin^9,10^ and animal-derived compounds such as lactoferrin and propolis.^11,12^

Given the close association between gut microbiota composition and susceptibility to *G. intestinalis* infection,^13,14^ probiotics (*live microorganisms that, when administered in adequate amounts, confer a health benefit on the host*^15^) represent a promising alternative strategy for the prevention and treatment of giardiasis. Indeed, the administration of probiotics has showed protective effect against a number of pathological conditions, including IBS,^16^ inflammatory bowel disease (IBD),^17^ metabolic syndrome^18^ and SARS-CoV2 infection,^19^ among others. However, there is a paucity of studies reporting the efficacy of probiotics in controlling *G. intestinalis* infection. To date, the efficacy of probiotics alone has only been demonstrated *in vitro*^20^ and *in vivo* in preclinical rodent models.^21–23^ Two clinical trials demonstrated the protective effect of probiotic *Enterococcus faecium* SF68 and *Saccharomyces boulardii*, when administered in combination with metronidazole therapy in dogs^24^ and humans,^25^ respectively. The protective effect of probiotics in *G. intestinalis* infection may involve different mechanisms, including stimulation of the host immune response to enhance the production of nitric oxide, IgA and IgG,^26–28^ secretion of bacteriocin^29^ or stimulation of host mucus production.^21,30^

The probiotic strain *Lactobacillus johnsonii* CNCM I-4884 (formerly classified as *Lactobacillus gasseri*) has been shown to significantly inhibit *G. intestinalis* proliferation in a murine model of giardiasis.^31^ In addition, we recently demonstrated that the protective effect of *L. johnsonii* CNCM I-4884 is mediated by its production of bile salt hydrolase (BSH) enzymes, which convert conjugated bile acids (BAs), major bile components essential for *G. intestinalis* growth, into free BAs, which are toxic to the parasite (Boucard et al., manuscript in preparation).

The main objective of this study was to use adaptive laboratory evolution to develop derivatives of *L. johnsonii* CNCM I-4884, with the aim of enhancing anti-*Giardia* properties of the strain. A collection of 12 mutants exhibiting increased resistance to BAs and reduced autolysis was generated and initially screened for their anti-*Giardia* activity on *G. intestinalis* trophozoite cultures. Three derivatives, M3, M5 and M11, were selected for further characterization of their growth, adhesion to intestinal cell lines, biofilm formation, morphology, BSH activity, and whole genome sequence. It is important to highlight that these derivatives were generated without molecular biology techniques and may thus be considered as food-grade microorganisms. The most promising candidate, M11, was also characterized at the proteomic level, and its protective effect was investigated *in vivo* in a murine model of giardiasis.

## Results

### *Screening of stress-resistant derivatives of* L. johnsonii *CNCM I-4884*

**Figure 1.**
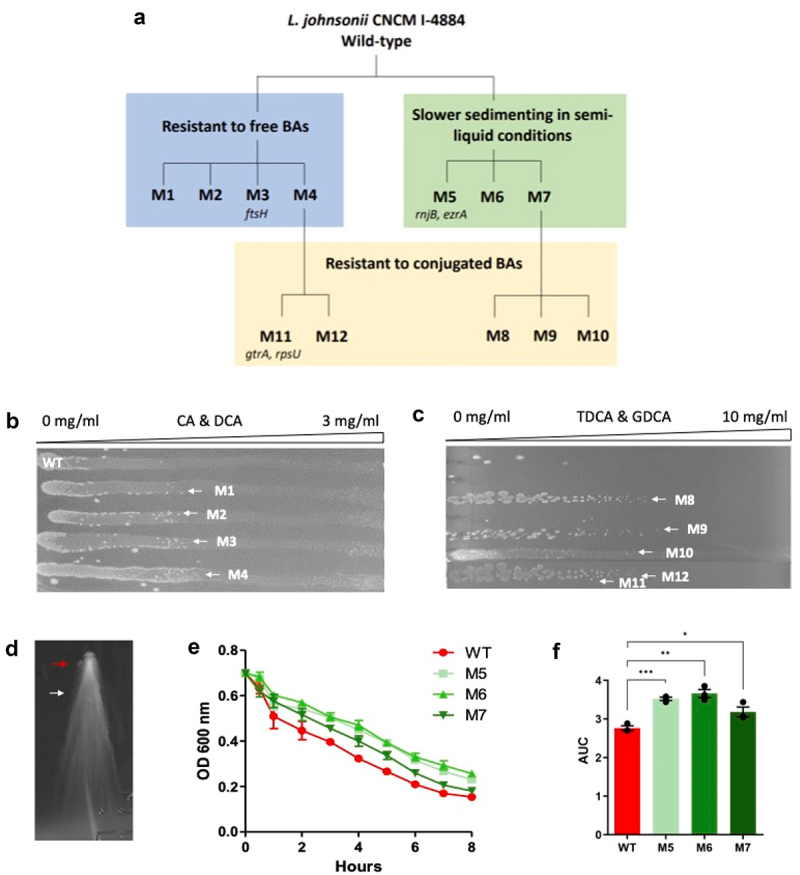
Screening and characterization of derivatives of *L. johnsonii* CNCM I-4884. (a) Schematic representation of the derivatives selection process. Refer to section “*whole genome sequencing*” for details on mutation identification. (b) Clones resistant to free BAs were selected using CA and DCA gradients. (c) Clones resistant to conjugated BAs were selected on TDCA and GDCA gradients. (d) Clones with altered sedimentation profiles were selected on THY + 0.03% agar; white arrow shows elongated wild-type colonies, red arrow points to slower sedimenting mutants with round colonies. (e) Autolysis was assessed by OD 600 nm in K₂HPO₄/KH₂PO₄ buffer + 0.05% triton X-100; data are shown as mean ± SEM (*n* = 3). (f) Area under curve of OD 600 nm. Asterisks indicate statistical significance compared to wild-type, as determined with unpaired *t-*test (***p < 0.001; **p < 0.01; *p < 0.05).

The strategy to generate stress-resistant derivatives involved three selective pressures, as shown in Figure 1a. Lactobacilli are naturally sensitive to BAs. It has been described that the production of BSH can confer enhanced resistance to BAs. Therefore, the main objective of this screening strategy was to generate derivatives with improved resistance to both free and conjugated BAs, potentially enhancing BSH production and improving persistence in the host. To do so, isolated clones of *L. johnsonii* CNCM I-4884 wild-type were grown on three successive gradients of MRS medium with increasing concentrations of cholic acid (CA) and deoxycholic acid (DCA). This lead to isolation of four clones, designated M1 to M4, that displayed increased resistance compared to wild-type. In the second screen, *L. johnsonii* CNCM I-4884 wild-type was grown on THY semi-liquid medium to isolate clones with modified surface properties. Under these conditions, single cell bacteria sediment and form prolonged colonies. In contrast, bacteria forming chains and aggregates are immobilized and do not sediment, and thus are easily distinguishable. Since bacterial chains often correspond to cell separation defects due to the robustness of their cell wall, such clones are expected to have reduced autolysis.^32^ Clones with delayed sedimentation were tested for resistance to autolysis in phosphate buffer containing 0.05% Triton X-100. Three derivatives (M5 to M7) showed significantly reduced autolysis compared to wild-type (*p <* 0.001; *p =* 0.002; *p =* 0.040, respectively). Finally, M1 to M7 candidates from the initial screens were grown on gradients of taurodeoxycholic acid (TDCA) and glycodeoxycholic acid (GDCA) to select derivatives resistant to conjugated BAs. This step yielded five additional resistant clones (M8 to M12) with increased tolerance to BAs compared to wild-type. Clones M8 to M10 were from autolysis-resistant M7, whereas M11 and M12 were from M4 derivative resistant to unconjugated BAs.

### *In vitro anti*-Giardia *activity*

**Figure 2.**
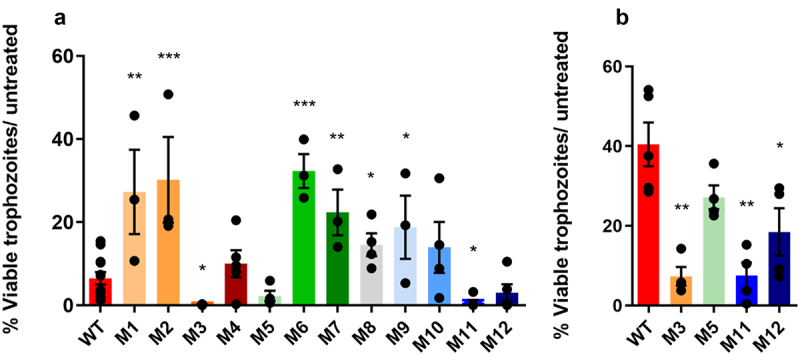
Growth inhibition of *G. intestinalis* trophozoites by culture supernatants of *L. johnsonii* CNCM I-4884 wild-type and derivatives relative to untreated parasite cultures, tested as undiluted (a) and 1/2 diluted (b) bacterial supernatants. Values are mean ± SEM (*n* = 4). Asterisks indicate statistical significance compared to wild-type, as determined with unpaired *t-*test (***p < 0.001; **p < 0.01; *p < 0.05).

To assess the anti-*Giardia* activity of *L. johnsonii* CNCM I-4884 wild-type and derivatives, *G. intestinalis* trophozoites were incubated with different concentrations of bacterial culture supernatants. After 22 h, viable trophozoites were counted and compared to untreated parasite cultures. Undiluted culture supernatants from all *L. johnsonii* strains reduced trophozoite viability (Figure 2a). Moreover, the M3 and M11 undiluted supernatants showed significantly higher levels of parasite growth inhibition than undiluted wild-type supernatant, with 0.1% and 0.0% viable trophozoites compared to 6.5% for wild-type (*p =* 0.032 and *p =* 0.028, respectively). In the presence of ½ diluted supernatant, M3 and M11 still showed a significant enhanced inhibitory activity, with 7.3% (*p =* 0.002) and 7.5% (*p =* 0.002) live trophozoites, respectively, compared to 40.5% for wild-type. M5 also showed increased inhibitory activity with 27.1% live trophozoites, but the difference with wild-type did not reach statistical significance (Figure 2b).

### Bacterial growth rates

**Figure 3.**
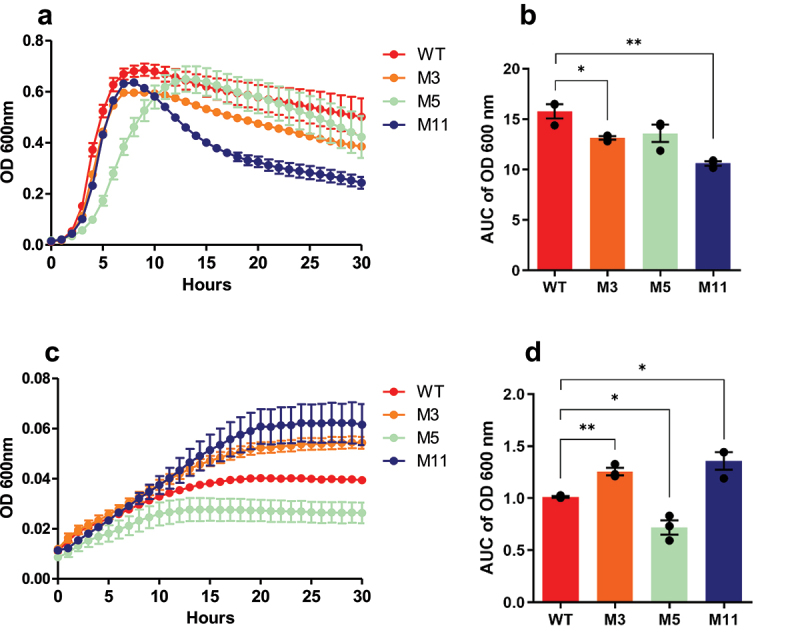
Growth rates of *L. johnsonii* CNCM I-4884 wild-type and derivatives in MRS medium (a) and area under curve (b) or MRS supplemented with a mix of conjugated BAs (1 mg/ml TDCA and GDCA) (c) and area under curve (d). Data are represented as mean ± SEM (*n* = 3). Asterisks indicate statistical significance compared to wild-type, as determined with unpaired *t-*test (**p < 0.01; *p < 0.05).

The M3, M5, and M11 derivatives, i.e., one from each screen, were selected for further phenotypic characterization relative to wild-type. First, their growth rates were assessed over 30 h at 37°C, in absence or presence of a mix of conjugated BAs (TDCA and GDCA). In MRS medium, M3 and M11 showed significantly reduced or retarded growth rate compared to wild-type (*p =* 0.022 and *p =* 0.002, respectively) (Figure 3a,b). However, in the presence of conjugated BAs, the growth rates of all three derivatives were significantly improved compared to wild-type (*p =* 0.003, *p =* 0.013 and *p =* 0.015, respectively) (Figure 3c,d).

### Adhesion to intestinal cell lines

**Figure 4.**
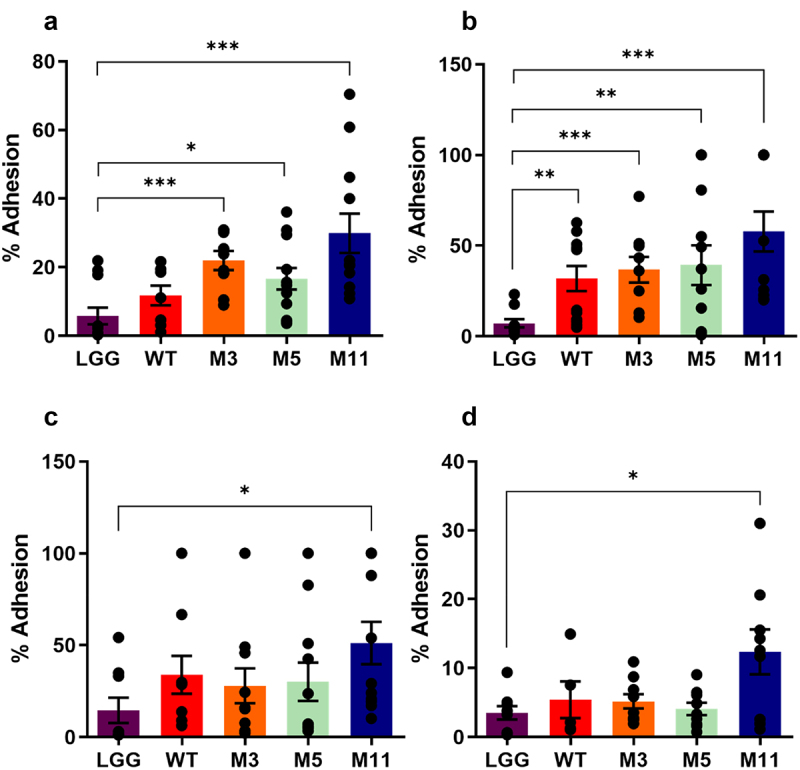
Adhesion of *L. johnsonii* CNCM I-4884 wild-type and derivatives to Caco-2 (a), HT-29 (b), HT-29 MTX (c) and T84 cell lines (d). Data are represented as mean ± SEM (*n* = 12). Asterisks indicate statistical significance compared to LGG, as determined with unpaired *t-*test (***p < 0.001; **p < 0.01; *p < 0.05).

Certain probiotic strains have the ability to adhere to the intestinal mucosa or the upper mucus layer, which may enhance their capacity to exert beneficial effects on the host. The adhesive properties of *L. johnsonii* CNCM I-4884 wild-type and derivatives were tested *in vitro* on four intestinal cell lines, using *Lacticaseibacillus rhamnosus* strain GG (LGG) as a positive control (Figure 4). The wild-type strain showed adhesion comparable to LGG on Caco-2, HT-29 MTX and T84 cell lines, and significantly higher adhesion on HT-29 cell line (*p* = 0.003). The derivatives showed similar or higher adhesion capacity than LGG for the four cell lines tested. Notably, M11 showed significantly improved adhesion to Caco-2 (*p* < 0.001), HT-29 (*p* < 0.001) HT-29 MTX (*p* = 0.030) and T84 (*p =* 0.019) cell lines compared to LGG.

### Biofilm formation

**Figure 5.**
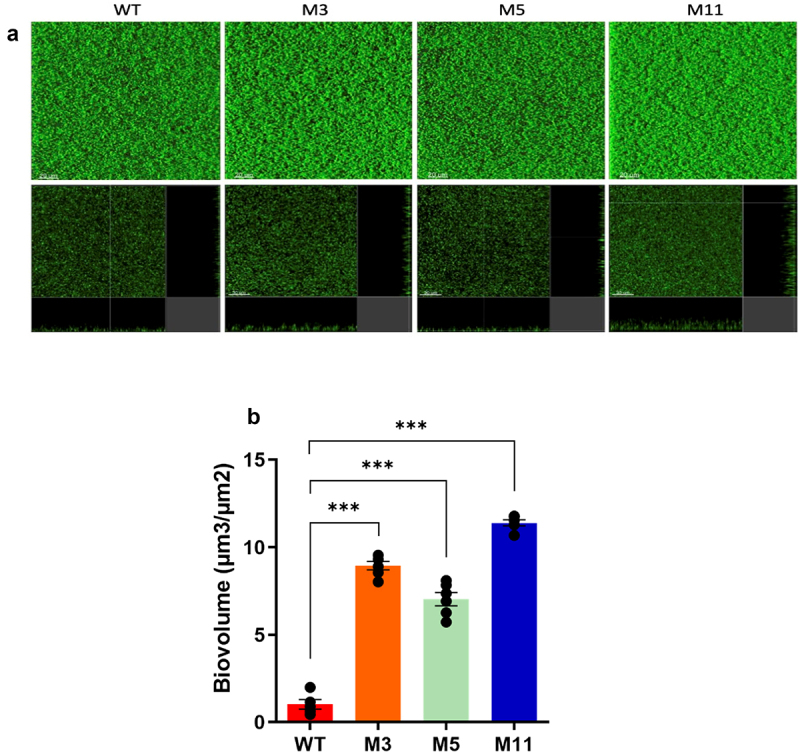
Biofilm formation by *L. johnsonii* CNCM I-4884 wild-type and derivatives. (a) 3D projections and section views of biofilm structure obtained from confocal z-stacks using IMARIS software; white scale bars represent 20 μm (b) quantification of biofilm biovolume. Data are presented as mean ± SEM (*n* = 6). Asterisks indicate statistical significance compared to wild-type, as determined with unpaired *t*-test (***p < 0.001).

Biofilms are bacterial communities of surface-attached bacteria embedded in an extracellular matrix. Through the gastrointestinal tract (GIT), biofilms grow naturally on both the epithelial surface and in the lumen as colonies attached to mucin and food particles.^33^ The ability of *L. johnsonii* CNCM I-4884 wild-type and derivatives to form biofilms *in vitro* was investigated using confocal microscopy. As shown in Figure 5, the wild-type strain formed an homogeneous biofilm that adhered effectively to the well surface. In contrast, M3, M5, and M11 derivatives formed thicker and denser mature biofilms, with a corresponding increase of biovolume (*p <* 0001; *p <* 0001 and *p <* 0001, respectively). Notably, M11 showed the highest biofilm formation, probably linked to its higher adhesive capacity, as shown in Figure 4. Consistent with previous findings, this increased adhesion and biofilm formation may provide M11 with a greater ability to persist *in vivo* by transiently colonizing the mucosal layer and/or intestinal epithelium, thus enhancing its probiotic effects.^34–36^

### TEM observations

**Figure 6.**
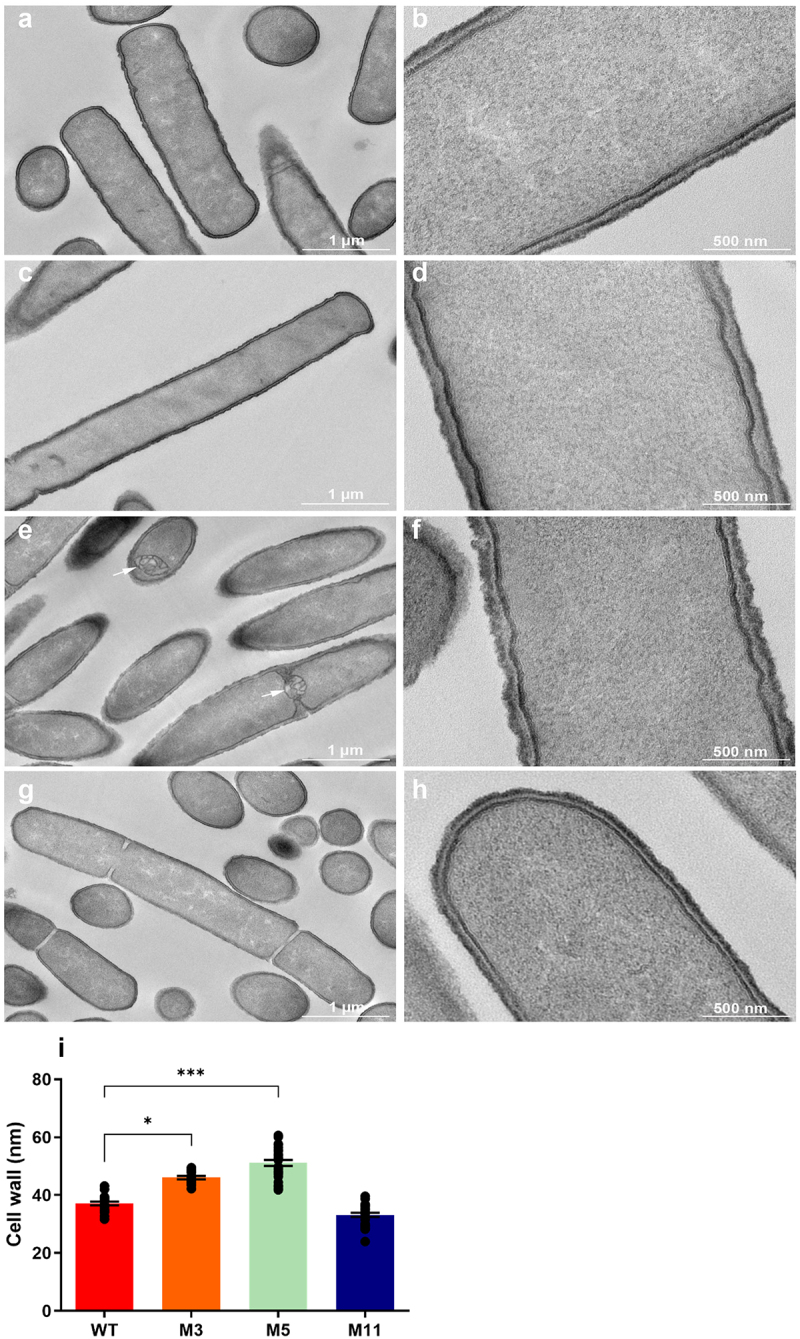
TEM observations of *L. johnsonii* CNCM I-4884 wild-type (a, b), and M3 (c, d), M5 (e, f) and M11 (g, h) derivatives, fixed in exponential growth phase. Inclusions (in M5) are indicated with an arrow. (i) Cell wall thickness. Data are represented as mean ± SEM (*n* = 15 to 30). Asterisks indicate statistical significance compared to wild-type as determined with unpaired *t-*test (***p < 0.001; *p < 0.05).

Given that some derivatives displayed altered sedimentation profile in semi-liquid medium and reduced autolysis, potentially indicating modified surface properties, the cell wall (CW) structure of *L. johnsonii* CNCM I-4884 wild-type and derivatives was investigated by TEM (Figure 6). M3 and M11 exhibited a morphology similar to the wild-type strain, with an average cell lenght of 9 µm. In contrast, variant M5 presented elongated cells (14 µm), with a thicker, irregular CW (45 nm) and numerous intracellular inclusions. Consistent with its altered sedimentation profile and decreased autolysis, M5 also presented an irregular CW which is significantly thicker than wild-type (*p <* 0.001).

### Whole genome sequencing

The complete genomes of M3, M5, and M11 derivatives were sequenced, assembled, and compared to the genome of *L. johnsonii* CNCM I-4884 wild-type^37^ to identify genetic differences. Overall, 1, 2, and 2 mutations were identified in the coding regions of M3, M5, and M11 genomes, respectively. The M3 genome revealed a missense mutation in *ftsH* gene, which encodes a membrane-anchored zinc metalloprotease with dual chaperone-protease activities. FtsH plays an important role in bacterial stress response mechanism, enabling rapid proteome adaptation to withstand harsh conditions and sudden environmental changes.^38–40^ In *Lactiplantibacillus plantarum*, FtsH expression is significantly upregulated in response to various stress conditions, including heat, oxidative and bile stress.^41^ FtsH has been proposed to impact the physicochemical properties of the cell surface, either directly or indirectly.^42^ In *Escherichia coli*, the challenges associated with obtaining a viable FtsH-null mutant suggest that FtsH is essential for survival in this species.^38^ Conversely, in Gram-positive bacteria, FtsH is not considered essential. Nonetheless, loss of FtsH function in both *L. plantarum* and *L. rhamnosus* leads to reduce growth rates under normal conditions, with the growth impairment becoming more pronounced under stress conditions.^43,44^ In contrast, a genetically modified *L. plantarum* overexpressing FtsH showed improved growth in the presence of bile.^42^ In M3, the mutation resulted in a replacement of an alanine (Ala) residue by a valine (Val) at position 53 within the periplasmic region of FtsH, a critical site for proper regulation of substrate selection. As shown in Figure 3, M3 growth is delayed under standard conditions but is improved in the presence of BAs. Furthermore, key residues within ATPase and protease domains of FtsH are conserved, indicating that the mutation should not compromise protein functionality. In M3, the FtsH mutation in the substrate recognition domain may enhance its substrate binding or proteolytic activity, resulting in improved resistance to BAs. In addition, indirect observations suggest a putative function of FtsH in modulating the CW architecture of *Lactococcus lactis*,^45^ which may explain the thicker CW observed in M3.

The M5 genome presents a mutation that introduces a premature stop codon in the *rnjB* gene encoding J2 ribonuclease. Ribonucleases J1 and J2 play roles in degradation of messenger RNA and maturation of non-coding RNA.^46,47^ The entire *rnjA* gene, encoding ribonuclease J1, remains intact in the M5 genome. These two enzymes are paralogs with conserved function; however, gene knockout in *Bacillus subtillis* has shown that RNase J1 (*rnjA*) is essential, whereas RNase J2 (*rnjB*) is not.^48^ This suggests that the loss of RnjB functionality in M5 may be compensated for by the presence of RnjA, and most probably would not affect M5 phenotype. A second mutation in M5 was identified in the *ezrA* gene, altering a glutamate (Glu) residue to an aspartate (Asp) at the C-terminal end of the protein. EzrA is a membrane-associated protein coordinating cell division and cell wall synthesis. During the cell cycle, the cell division protein FtsZ forms a ring structure that determines the site of nascent division. EzrA acts as a negative regulator of FtsZ, modulating both the frequency and precise position of FtsZ ring formation.^49^ This mutation could be responsible for the elongated cell shape and multiple inclusions at polar and medial sites observed in TEM images (Figure 6), a phenotype similar to that described in knockout mutants of *Bacillus subtilis* and *L. plantarum*.^49–51^

The M11 genome contains a missense mutation in the *gtrA* gene, resulting in a change of serine (Ser) to a tyrosine (Tyr) at position 114. The *gtrA* gene encodes a putative flippase of the GtrA superfamily, involved in the production of branched glucan homopolysaccharide.^52^ Exopolysaccharides (EPS), which protect bacterial surfaces and facilitate host interactions, create a more hydrophilic and less negatively charged surface, reducing cell self-aggregation. Compared to wild-type, M11 shows increased self-aggregation in liquid culture (data not shown), suggesting a loss of function in EPS biosynthesis.^52,53^ Studies on EPS production have shown contradictory effects on stress resistance. For example, in *L. johnsonii* FI9785, reduced EPS production led to lower survival under stress conditions, whereas in *Bifidobacterium longum*, it improved acid tolerance.^53,54^ Furthermore, decreased EPS production in *L. johnsonii* FI9785 and *L. johnsonii* La1 strains increased their adhesion to chicken gut explants^53^ and improved their persistence in the murine GIT.^55^ This suggests that M11, with altered EPS production, might also show better persistence in the GIT than the wild-type counterpart. Finally, the M11 genome also contains a missense mutation in *rpsU* gene, resulting in a change from a lysine (Lys) to an asparagine (Asn) at position 44. The *rpsU* gene encodes the 30S ribosomal protein S21p. Consistent with M11 stress-resistant phenotype, variants of *Listeria monocytogenes* affected in *rpsU* also presented an enhanced resistance to various stresses, including acid, cold and heat stress.^56^ As with M11, *L. monocytogenes* variants showed slower growth rate and increased adhesion to Caco-2 cells compared to the wild-type.^57^

### Bile salt hydrolase activity

**Figure 7.**
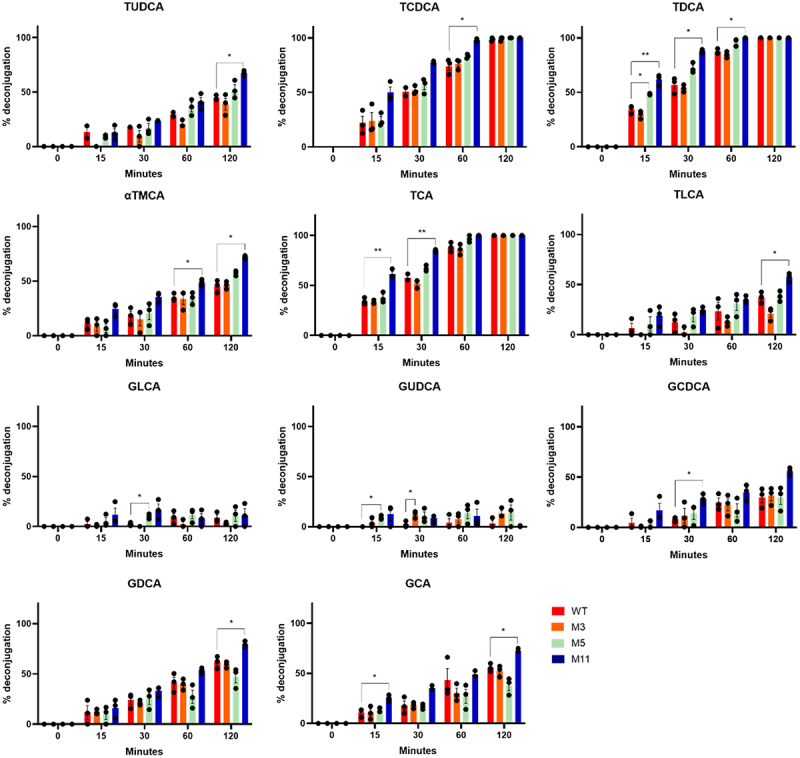
Bile acids (BAs) deconjugation activity of supernatant samples issued from cultures of *L. johnsonii* CNCM I-4884 wild-type and derivatives, after various incubation times. Data are represented as mean ± SEM (*n* = 3). Asterisks indicate statistical significance compared to wild-type, as determined with unpaired *t-*test (**p < 0.01; *p < 0.05). TUDCA : tauroursodeoxycholic acid; TCDCA : taurochenodeoxycholic acid; TDCA : taurodeoxycholic acid; αTMCA : tauromuricholic acid; TCA : taurocholic acid; TLCA : taurolithocholic acid; GLCA : glycolithocholic acid; GUDCA : glycoursodeoxycholic acid; GCDCA : glycochenodeoxycholic acid; GDCA : glycodeoxycholic acid; GCA : glycocholic acid.

To determine whether the higher anti-*Giardia* activity observed in the stress-resistant derivatives was related to higher BSH production, the strains were exposed to a panel of tauro- and glyco-conjugated BAs. BSH activity was assessed by measuring the levels of conjugated and free BAs by HPLC MS/MS. All four strains, including the wild-type and the three derivatives, demonstrated the ability to hydrolyze a wide range of conjugated BAs commonly found in humans and other mammals (Figure 7). In particular, M11 showed significantly higher hydrolytic activity than wild-type for all tauroconjugated BAs tested, as well as for several glycoconjugated BAs, including GCDCA, GDCA, and GCA. The higher BSH activity of M11 is consistent with its increased BAs resistance and higher anti-*Giardia* activity observed *in vitro* (Figures 1,2). Altogether, these results led to the selection of M11 candidate for further characterization.

### Proteomic analysis

**Figure 8.**
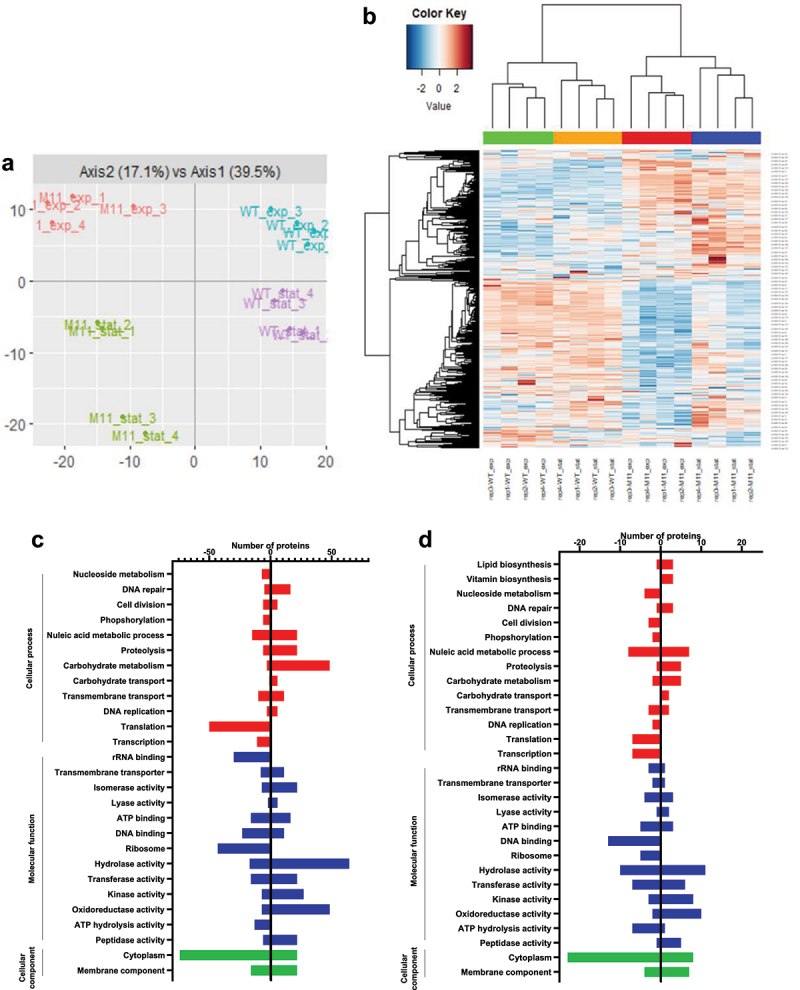
Proteomic analysis of *L. johnsonii* CNCM I-4884 wild-type and M11 derivative. (a) Principal component analysis. (b) Global heatmap representation of protein abundances significantly different in wild-type and M11 (*n*=4, ANOVA, adjusted *p<*0.05). Each row represents a differentially expressed protein, while each column represents a sample. (c) Functional classification based on GO enrichment analysis of differentially expressed proteins in exponential or (d) stationary growth phase.

In order to elucidate the impact of M11 mutations on its metabolism, wild-type and M11 proteomes were analyzed by LC-MS/MS in exponential and stationary culture growth phases. Based on the identified peptides, a total of 746 proteins were identified in wild-type and M11, representing 41.35% of the theoretical proteome. Data treatment resulted in 660 valid proteins quantified by XIC (extracted ion chromatograms). Principal component analysis (PCA) based on the 660 proteins revealed that samples were grouped by strain and growth phase (Figure 8a). For each growth phase, wild-type and M11 proteomes were compared to identify proteins with significantly different abundances. In exponential growth phase, 198 proteins were differentially expressed in M11 compared to wild-type, representing 26.5% of total proteins detected, among which 51 proteins were up-regulated and 147 were down-regulated with a log2 fold change ≥2 (*p ≤* 0.05). In stationary growth phase, 104 proteins were differentially abundant in M11 compared to wild-type, representing 13.9% of the proteins detected, among which 52 proteins were more abundant in M11 and 52 proteins were less abundant in M11 compared to wild-type. Proteins with statistically different abundance in the two strains are represented in a heatmap using hierarchical clustering with Euclidean distances (Figure 8b). The biological replicates grouped together, and the samples were clearly resolved in two main clusters corresponding to the two strains.

The Cluster of Orthologous Groups (COG) database was used to elucidate the function of differentially abundant proteins (Figure 8c,d). During exponential growth phase, the major molecular functions upregulated in M11 were isomerase, kinase, hydrolase and oxidoreductase activities involved in carbohydrates metabolism, nucleic acid metabolism, proteolysis and DNA repair. In contrast, downregulated molecular functions in M11 included mainly structural components of ribosome, rRNA and DNA binding, involved in transcription and translation. The same trend was observed in stationary growth phase samples.

The individual abundance of proteins belonging to the main cellular processes significantly different in M11 compared to wild-type are presented in Figure 9. Interestingly, BSH450 and BSH1011 enzymes were among the most overexpressed proteins in M11 compared to wild-type, with 3.11 and 3.94 log2 fold change, respectively, in exponential growth phase and with 2.63 and 3.18 log2 fold change, respectively, in stationary growth phase, which is consistent with the enhanced BSH activity of M11 observed in Figure 7. In addition, formyl-CoA transferase and oxalyl-CoA carboxylase, which are involved in oxalate metabolism, were among the most overexpressed proteins with 6.15 and 4.93 log2 fold change in exponential growth phase and overexpressed by 3.36 and 3.72 log2 fold change in stationary growth phase respectively.

**Figure 9.**
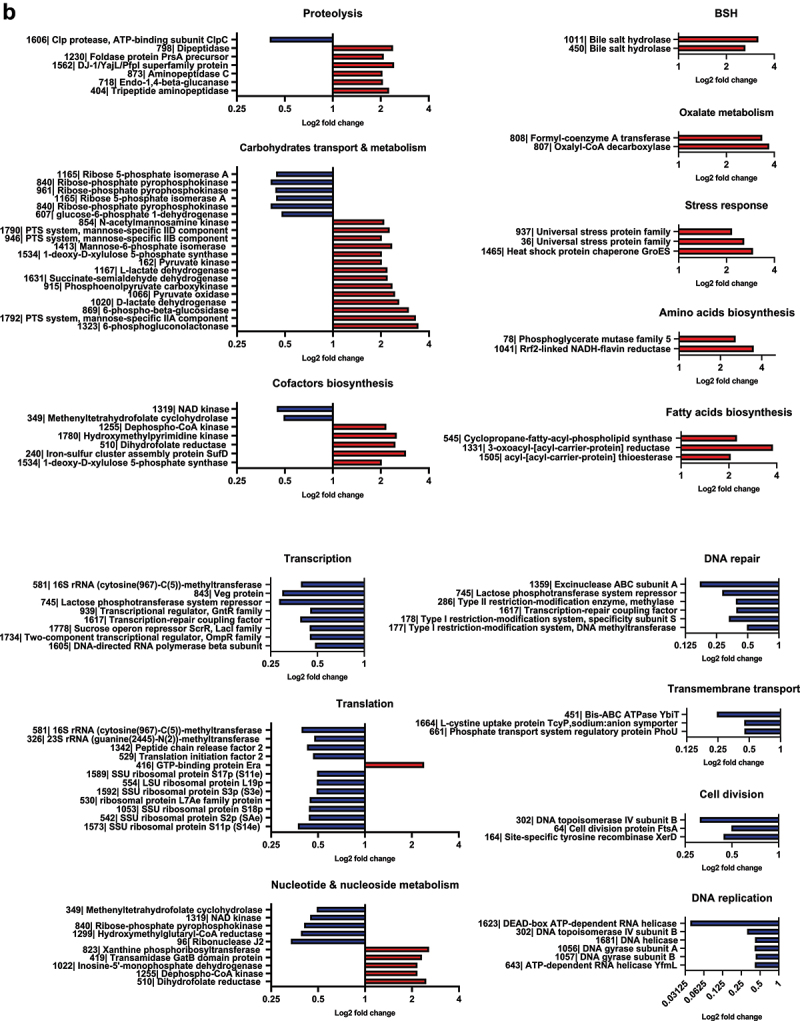
(Continued).

In exponential growth phase, M11 samples showed a higher abundance of proteins involved in glycolysis, pentose phosphate pathway, pyruvate, nucleotide and nucleoside metabolisms. M11 also showed an increased abundance of proteins involved in cofactor biosynthesis, such as thiamine, folate and iron-sulfur (Fe-S) cluster biosynthesis. In contrast, M11 showed a significant downregulation of proteins involved in transcription and transcriptional regulation, including 48 ribosomal proteins and the mutated S21p protein. Other downregulated proteins were involved in transmembrane transport, translation, DNA replication, cell division and DNA and protein repair.

In the stationary growth phase, most of the altered functions were similar to those observed in exponential growth phase. In addition, M11 samples also showed a higher abundance of proteins involved in general stress response and fatty acid and amino acid biosynthesis compared to wild-type samples. Interestingly, the mutated GtrA protein showed no difference in abundance between M11 and wild-type in either growth phases assays.

**Figure 9.**
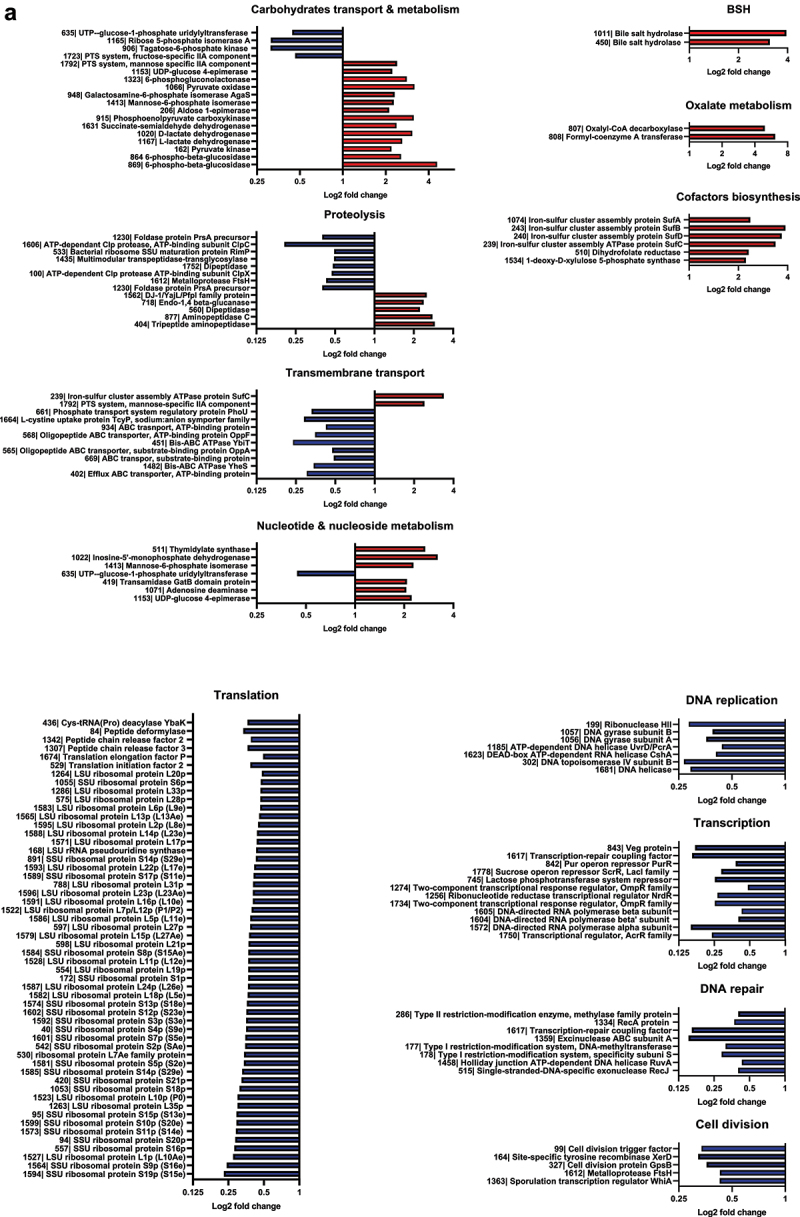
Fold-change of proteins with significant abundance change (ANOVA, adjusted *p<*0.05) between *L. johnsonii* CNCM I-4884 wild-type and M11 derivative, belonging to the main functional categories in (a) exponential and (b) stationary growth phase.

### *In vivo anti*-Giardia *activity*

**Figure 10.**
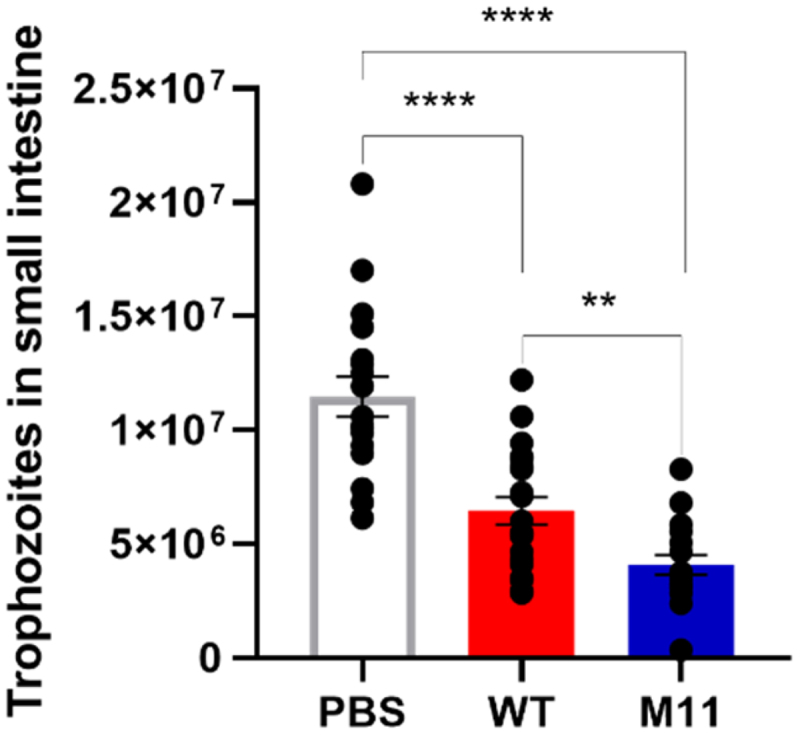
Reduction of *G. intestinalis* trophozoite load in the small intestine of OF1 mice treated daily with either PBS, *L. johnsonii* CNCM I-4884 wild-type or M11, 6 d after *Giardia* infection challenge. Values are mean ± SEM (*n*=18). Asterisks indicate statistical significance, as determined with unpaired *t-*test (****p<0.0001; **p<0.01).

The anti-*Giardia* activity of *L. johnsonii* CNCM I-4884 wild-type and M11 were investigated *in vivo* in a murine model of giardiasis. Five-days-old OF1 mice were treated daily by probiotic gavage for 10 d and challenged with *G. intestinalis* trophozoites 5 d after the first probiotic administration. Six days after infection, *G. intestinalis* trophozoites were quantified in the small intestine (Figure 10). Both probiotic treatments significantly reduced trophozoite load compared to PBS control. In addition, M11 enhanced trophozoites growth inhibition compared to wild-type, with a respective reduction of parasite load of 43.8% and 64.4% compared to PBS control. As BSH activity could affect weight gain in young animals,^58^ we monitored daily weight gain; however, no difference was observed between mice receiving probiotic treatments compared with control mice (data not shown).

## Materials and methods

### Bacterial strains

*Lactobacillus johnsonii* CNCM I-4884 wild type and its derivatives (M1 to M12) as well as *Lacticaseibacillus rhamnosus* strain GG (LGG, ATCC 53103) were stored at −80°C in Man Rogosa Sharpe (MRS) broth (Difco, France) with 20% glycerol until further analysis. Strains were routinely grown in MRS at 37°C under microaerobic conditions.

### Intestinal cell lines

The human colon adenocarcinoma cell lines Caco-2 and HT-29 were obtained from the American Type Tissue Collection (ATCC® HTB-37™ and ATCC® HTB-38™ respectively), the T84 cell line was obtained from the European Collection of Authenticated Cell Cultures (ECACC 88021101), and the mucus-producing HT-29 MTX cell line was obtained from the Sloan Kettering Memorial Cancer Center (SKMCC). All cell lines were grown in Dulbecco modified Eagle’s medium (DMEM) supplemented with 1% penicillin/streptomycin and 20%, 10%, 10% and 6% heat-inactivated fetal calf serum (FCS), respectively. The culture medium of Caco-2 cells was supplemented with 1% non-essential amino acids. Cell lines were maintained at 37°C in a humidified atmosphere with 10% CO_2_.

### *Selection of stress-resistant derivatives of* L. johnsonii *CNCM I-4884*

#### Resistance to free BAs

Overnight cultures of isolated clones of *L. johnsonii* CNCM I-4884 were streaked on MRS agar plates with a gradient (from 0 up to 1 mg/ml) of free BAs (mix of cholic acid and deoxycholic acid, Sigma, USA) and incubated at 37°C under anaerobic conditions. After 48 h, clones with the highest resistance were streaked successively on MRS agar plates with gradients of free BAs up to 2 mg/ml and 3 mg/ml. Clones obtained after the third passage were considered resistant to these BAs.

#### Resistance to autolysis

Mutants with altered sedimentation profiles were isolated using Todd Hewitt broth supplemented with 0.5% yeast extract (THY) with a low agar concentration (0.03%) as previously described.^32^ Fifty ml flasks of THY semi-liquid medium were inoculated with 10 µl of a diluted *L. johnsonii* CNCM I-4884 overnight culture, corresponding to approximately 5 to 10 cells/flask, and incubated at 37°C for 14 d until appearance of slow sedimenting clones (see Figure 1d). Clones with different sedimentation profiles were then isolated on MRS agar plates. Overnight cultures of the recovered clones were washed twice in Phosphate Buffered Saline (PBS), diluted to an optical density at 600 nm (OD_600_) = 0.7 in 50 mM K_2_HPO_4_/KH_2_PO_4_ pH 7 + 0.05% Triton X-100 (Merck, Germany) and incubated at 37°C. Lysis was monitored by measuring the decrease in OD_600_ in the cell suspensions.

#### Resistance to conjugated BAs

Overnight cultures of clones resistant to autolysis and free BAs were streaked on MRS agar plates with a gradient (from 0 up to 10 mg/ml) of conjugated BAs (mix of sodium taurodeoxycholate and sodium glycodeoxycholate, Sigma, USA) and incubated at 37°C under anaerobic conditions. Clones obtained after the last screening were considered resistant to these BAs.

### *In vitro anti*-Giardia *activity*

The anti-*Giardia* activity of *L. johnsonii* CNCM I-4884 wild-type and derivatives was assessed as previously described.^31^ Briefly, bacterial strains were grown in modified Keiser’s TYI-S-33 (KM) medium supplemented with 10% FCS for 20 h under anaerobic conditions. Filtered bacterial supernatants were co-incubated with *G. intestinalis* WB6 trophozoites at 1.33 × 10^5^ cells/ml in KM at pH 6.0 supplemented with 10% FCS and 0.6 mg/ml bovine bile at a volumetric ratio of 1:3 at 37°C under anaerobic conditions. After 22 h, samples were ice-chilled for 10 min and trophozoite load was determined using hemocytometer (flagellar mobility was used to assess parasite viability).

### Adhesion to intestinal cell lines

Caco-2, HT-29, HT-29 MTX and T84 cells were seeded at 1 × 10^5^ cells/well in 24-well culture plates and grown for 21 d. Antibiotics were removed from the culture medium 24 h prior to co-incubation with bacteria. Overnight bacterial cultures were washed twice with PBS and resuspended in antibiotic-free cell culture medium and added to each well at 2 × 10^8^ CFU/ml. After 1 h incubation, cells were washed twice with PBS, adherent bacteria were detached from the cells using 0.05% Triton X-100 (Merck, Germany) and enumerated on MRS agar plates. The results are expressed as the percentage of bacteria adhering to the intestinal cells. LGG strain was used as a positive control.

### Biofilm formation

Overnight cultures of *L. johnsonii* CNCM I-4884 wild-type and derivatives were used to inoculate MRS in 96-well microplates (1:100). Bacteria adhered for 1 h at 37°C and were then washed twice with fresh medium. After 24 h incubation at 37°C, the obtained biofilms were stained with SYTO 9 (Life Technologies). After 30 min incubation, images were acquired with a Leica SP8 confocal laser scanning microscope (CLSM, Leica Microsystems). The emitted fluorescence signal was collected on a hybrid detector in the 500–550 nm range after excitation at 488 nm with an Argon laser set at 20% of its maximum intensity with a z-step of 1 µm and at 600 hz. Simulated 3D fluorescence projections were generated using IMARIS 9.1 software and biomass was calculated from raw images using Eclipse software.

### Transmission electron microscopy (TEM)

Exponential cultures of *L. johnsonii* CNCM I-4884 wild-type and derivatives were washed twice with PBS and fixed with 2% glutaraldehyde in 0.1 M sodium cacodylate buffer at pH 7.2 for 1 h at room temperature. The samples were then counterstained and analyzed as described in.^59^

### BSHs activity

*L. johnsonii* CNCM I-4884 wild-type and derivatives were cultured in KM medium adjusted to pH 6.0 and supplemented with 10% FCS for 8 h at 37°C under anaerobic conditions. BA concentrations were measured by HPLC-MS/MS as previously described.^60^ Experiments were stopped at various time points (0, 15, 30, 60, and 120 min) for each sample. We utilized a Sciex 5500Q-Trap mass spectrometer coupled with a Shimadzu LC-20ADXR dual binary flow HPLC system and a Kinetex 5 µm, 100 × 2.1 mm, C18 100 Å column (Phenomenex). Direct enzymatic activities were assessed by observing the decrease in conjugated BAs and the corresponding increase in unconjugated BAs.

### Whole genome sequencing and identification of mutations

Genomic DNA extraction, sequencing, and annotation were carried out as previously described.^37^ The genomes of M3, M5 and M11 derivatives were compared to the wild-type genome using the PATRIC variation analysis service. For each mutant strain, Illumina reads were mapped directly against the reference wild-type genome sequence using the BWA-MEM strict aligner. Single nucleotide polymorphisms (SNPs) and short insertions or deletions were identified using the FreeBayes method, and the effects of these SNPs were predicted with SNPeff.

### Proteomic analysis

#### Protein extraction

Cell pellets from exponential and stationary cultures of *L. johnsonii* CNCM I-4884 wild-type and M11 were resuspended in PBS supplemented with protease inhibitor (Roche, Germany). After homogenization with 0.10 to 0.25 mm diameter glass beads (Fisher Scientific) using a Precellys Evolution homogenizer (Bertin Technologies, France) for 3 × 30 s at 6,400 rpm, the suspensions were centrifuged for 5 min at 13,000 g and the supernatants were collected. Ten mg of each protein extract were separated by a short migration in a polyacrylamide gel. Gel pieces with total protein extracts were collected and incubated with 10 mm DTT at 56°C for 30 min. Samples were then supplemented with 55 mm iodoacetamide and incubated in the dark at room temperature for 45 min (alkylation). Subsequently, 50 mm NH_4_CO_3_ and 50% acetonitrile (ACN) were added to each sample. After 15 min, gel pieces were incubated with 100% ACN. Next, liquid was removed and 100 ng trypsin (Promega) was added, and samples were incubated first at 4°C for 15 min and then overnight at 37°C. Samples were first incubated with 50 mm NH_4_CO_3_ for 10 min, followed by 15 min incubation with 0.5% trifluoroacetic acid (TFA) and 50% ACN. Samples were then dried and resuspended in 0.08% TFA and 2% ACN.

#### LC-MS/MS analysis

The LC-MS/MS method was adapted from.^61^ MS analyses were performed on a Dionex U3000 RSLC coupled to an Orbitrap Fusion™ Lumos™ Tribrid™ mass spectrometer (Thermo Fisher Scientific). A 2 μl sample was loaded at 20 μl/min on a precolumn (μ-Precolumn, 300 μm i.d × 5 mm, C18 PepMap100, 5 μm, 100 Å, Thermo Fisher) and washed with loading buffer. After 3 min, the precolumn cartridge was connected to the separating column (Acclaim PepMap®, 75 μm × 500 mm, C18, 2 μm, 100 Å, Thermo Fisher). Buffer A consisted of 0.1% formic acid in 2% acetonitrile and buffer B of 0.1% formic acid in 80% acetonitrile. The gradient was executed at 250 nl/min with a linear gradient from 2% to 40% of buffer B for 115 min, and one run took 147 min including the generation phase (98% of buffer B). LC-MS/MS analysis was performed utilizing a nanospray ionization source and the eluted peptides were ionized by applying 1,6 kV in positive mode. MS scans were performed at 120,000 resolution, m/z range 400‒ 1,500 Da. MS/MS analysis was performed in a data-dependent mode, with a top speed cycle of 3 s for the most intense double or multiple charged precursor ions. Ions in each MS scan over threshold 50,000 were selected for fragmentation (MS2) by higher energy collisional dissociation (HCD) at 30% for identification and detection in the orbitrap followed by top speed MS2 fragment ions. Precursors were isolated in the quadrupole with a 1.2 m/z window and dynamic exclusion within 10 ppm during 80 s was used for m/z-values already selected for fragmentation. The AGC targets were fixed as custom and standard for MS and MS/MS, respectively.

#### Proteins identification and quantification

A FASTA format database was used from *L. johnsonii* CNCM I-4884 WT genome (1809 entries,^37^). A database of common contaminants was also used for the analysis. Database searches were performed using the i2MassChroQ (version 0.4.62, http://pappso.inrae.fr/) with one possible missed cleavage. Carboxyamidomethylation of cysteine residues and oxidation of methionine residues were set to “static” and “possible” modifications, respectively. Precursor and fragment mass tolerance was 10 ppm. Identified proteins were filtered and grouped using i2MassChroQ. Data filtering was achieved according to a peptide *E-*value <0.01, protein log (E-value) < −4 and to a minimum of two identified peptides per protein. Relative quantification of protein abundances was performed using extracted ion chromatograms (XIC) method defined as the sum of MS1 intensities of all peptides associated with a protein. Data post-processing and statistical analysis were performed by using the R package4 MCQR 0.4.3 as previously described.^62^

### *In vivo anti*-Giardia *activity*

The anti-*Giardia* activity of *L. johnsonii* CNCM I-4884 wild-type and M11 was investigated *in vivo* as previously described.^31^ Bacterial strains (5 × 10^8^ CFU in PBS with 15% glycerol) were administered daily by intragastric gavage to 5 d old OF1 mice (Charles River, France) for 10 d. Control animals received PBS with 15% glycerol. Mice were then challenged with 1 × 10^5^ *G. intestinalis* WB6 trophozoites at 10 d of age by intragastric gavage. Mice were euthanized by cervical dislocation at 16 d of age. The small intestine was opened, resuspended in 5 ml ice-chilled PBS and mixed thoroughly. Trophozoites were enumerated using a hemocytometer. Protocols were conducted in accordance with the institutional guidelines approved by the local ethical committee and the Ministère de l’Education Nationale, de l’Enseignement Supérieur et de la Recherche, France (APAFIS #37444–2022050516561397).

### Statistical analysis

Statistical differences were determined using ANOVA and unpaired *t-*tests. All statistics were performed using GraphPad Prism (version 9.00) and values of *p* less than 0.05 were considered to be statistically significant.

## Discussion

In this study, we employed an adaptive evolution strategy to generate stress-resistant derivatives of *L. johnsonii* CNCM I-4884, with the aim of improving both BSH production and anti-*Giardia* activity in the strain. Adaptive evolution has been widely used to generate strains with phenotypes-of-interest, such as improved production of metabolites, like gamma-aminobutyric acid^63^ as well as enhanced tolerance to acid stress,^64,65^ temperature,^66^ antibiotic,^67^ or high NaCl concentrations.^68^ Here, this approach generated a collection of 12 stress-resistant derivatives, which were further evaluated at phenotypic and genetic levels. The candidate M11 showed the most marked phenotype, with a significantly improved anti-*Giardia* activity *in vitro* correlated with improved BSH activity toward two types of conjugated BAs. Although adaptive evolution has proven to be highly effective, it may result in significant genome rearrangements, including large deletions previously reported.^66^ Here, the presence of mutations affecting a single nucleotide was confirmed by whole genome sequencing. Two SNPs were identified in M11 genome relative to wild-type strain. The first mutation was identified in a gene encoding a putative flippase with homology to the GtrA superfamily involved in EPS biosynthesis. The reduced expression of EPS may be responsible for the enhanced auto-aggregative phenotype and adhesion to intestinal epithelial cell lines of M11, conferring the mutant a selective advantage for GIT colonization.^53–55^ Modifications in cell wall structure have been previously described in other laboratory adapted strains in the presence of stress.^69,70^ A second mutation was identified in *rpsU* gene encoding the 30S small subunit ribosomal protein S21p. Variants of *L. monocytogenes* mutated in the *rpsU* gene showed upregulation of the general stress factor Sigma B and SigB regulon members which are associated with a stress-resistant phenotype.^71^ Through proteomic analysis, we identified a number of differentially expressed proteins in M11 compared to wild-type. Similar to what has been observed in *L. monocytogenes* variants, SigB was significantly increased in M11 compared to wild-type, with 2.38 and 2.20 log2 fold change in exponential and stationary growth phases, respectively. This result suggests that the upregulation of general stress response by SigB may be responsible for M11 improved BAs resistance. The activation of a systemic stress defense response via SigB is energetically costly, resulting in a negative impact on growth. Indeed, M11 showed an altered growth rate compared to wild-type in standard conditions. However, when cultured in the presence of BAs, the growth of M11 was significantly improved, indicating a trade-off between stress resistance and fitness, as observed in previous studies.^65,71,72^ The impaired growth rate of M11 may also be attributed to the competition between SigB and housekeeping SigA for the RNA polymerase, with the latter responsible for the transcription of growth-related genes.^71^ Moreover, the activation of SigB may be responsible for the enhanced biofilm formation of M11, as reported in *L. monocytogenes*^73^ and *B. subtilis*.^74^

Furthermore, the comparative analysis of M11 and wild-type proteomes revealed a higher abundance of proteins involved in glycolysis, the pentose phosphate pathway and pyruvate metabolism. Previous studies have reported up-regulation of these pathways in other *Lactobacillus* species in response to acid stress^75–78^ and bile stress.^79–84^ This has been showed to result in increased ATP production, thereby providing the strain with sufficient energy to meet the elevated energy demands and the stress associated with the adaptation to BAs. The overexpression of L- and D-lactate dehydrogenase resulted in increased lactate and ATP production, as well as NAD+ regeneration, which collectively maintained redox balance during BAs stress. M11 also presented an increased abundance of formyl-CoA transferase and oxalyl-CoA carboxylase, involved in oxalate metabolism. This could represent an alternative energy source in the absence or depletion of a carbohydrate source^85^ and is important for bile stress resistance and GIT colonization.^82,86^ M11 also showed an enrichment of proteins with predicted peptidase activity. The overexpression of the proteolytic system is commonly observed in lactobacilli in response to acid and bile stress, in order to provide amino acids for the synthesis and repair of proteins damaged by stress.^78,81,87,88^ In addition, M11 exhibited a greater abundance of proteins involved in co-factor biosynthesis, particularly the SUF machinery responsible for the assembly of Fe-S clusters. These clusters are essential for the correct activity of housekeeping proteins and transcriptional regulators under stress conditions.^89^ A role of the SUF machinery has been also described in stress resistance and biofilm formation.^90^ In addition, M11 overexpressed proteins with oxidoreductase activity, which is consistent with previous studies.^81,83^ In addition to improving tolerance to bile-induced oxidative stress, the NADH-dependent oxidoreductase results in the recycling of ATP and doubles the ATP yield from carbohydrate metabolism, thereby amplifying the energy generation.^79^

Of particular interest is the overexpression of BSH450 and BSH1011 in M11 compared to wild-type. These findings are corroborated by the increased deconjugated activity of the M11 mutant toward both tauro- and glyco-conjugated substrates. These results align with a previous transcriptomic study which reported increased *bsh* expression in a variant of *L. monocytogenes* inactivated in *rpsU* gene.^55^ Moreover, additional research has indicated that the overexpression of BSH enzymes in response to bile stress has a favorable impact on adhesion and GIT persistence in other *Lactobacillus* species.^82,87,91,92^

The majority of down-regulated proteins in M11 are involved in cell division, DNA replication, transcription and translation, which is consistent with the decreased growth rate of M11 compared to wild-type observed in MRS medium. Surprisingly, proteins with chaperone activity and ABC transporters were also less abundant in M11 relative to wild-type. These findings contradict previous studies reporting an increase of these activities in response to stress in several *Lactobacillus* species, as they play an important role in the recycling of damaged proteins and the efflux of bile stress-related compounds.^80,87,88,93–95^

In stationary phase, M11 samples showed a further increase in proteins involved in the biosynthesis of unsaturated and cyclopropane fatty acids (CFA). Previous studies have reported increased CFA biosynthesis in lactobacilli in response to bile stress, preserving membrane integrity and counteracting proton influx.^93,95–97^ As described for other *Lactobacillus* species in response to stress, proteins involved in serine and lysine biosynthesis are more abundant in M11 compared to wild-type.^75,84,87,98^ It has been suggested that the increased lysine biosynthesis may facilitate lysinylation of membrane lipids and thus improve stress-resistance by altering the surface charge to repulse cationic bile compounds.^84,87^ Furthermore, M11 displayed increased abundance of universal stress proteins, consistent with previous observations in response to stress.^75,79,81^ It was also found that the universal stress protein UspA was upregulated in M11 strain. Of note, this protein has been reported to play a role in biofilm formation, and may be responsible for the enhanced biofilm biovolume of M11.^99,100^

Finally, the inhibitory activity of M11 was investigated *in vivo* in a suckling murine model of giardiasis. Mice treated with M11 exhibited a significantly higher reduction in parasite burden in the small intestine compared to those treated with wild-type, confirming the results observed *in vitro* on *Giardia* trophozoites. These findings substantiated the correlation between increased BSH activity and increased anti-*Giardia* activity both *in vitro* and *in vivo*.

A potential limitation of this study is the absence of complemented strains to confirm the role of RpsU in stress resistance and BSH activities. The whole genome sequencing of M11 helps to mitigate this limitation as it provides the complete genetic context of the mutations. Another limitation of this study is the absence of a negative control in the murine experiment. Further studies could benefit from the addition of a treatment group with a non BSH-producing strain or the administration of BSH inhibitors^101,102^ in order to confirm the role of these enzymes in the anti-*Giardia* activity of the strains.

In conclusion, this work provides evidence that adaptative evolution, resulting in the incorporation of single amino acid substitutions in GrtA and RpsU, has enhanced the stress-resistance, BSH production and consequently anti-*Giardia* activity of *L. johnsonii* CNCM I-4884 both *in vitro* and *in vivo*. Overall, our study describes an evolutive approach to develop more robust food-grade derivatives from wild-type probiotic strains, significantly enhancing their beneficial properties.
